# The Need for Education and Clinical Best Practice Guidelines in the Era of Direct-to-Consumer Genomic Testing

**DOI:** 10.2196/21787

**Published:** 2020-12-08

**Authors:** Madeleine Myers, Cinnamon Bloss

**Affiliations:** 1 University of California San Diego La Jolla, CA United States

**Keywords:** personal genome testing, direct-to-consumer, primary care, patient-physician relationship, medical education

## Abstract

Many people share the results of their direct-to-consumer personal genomic testing (DTC-PGT) within the primary care setting, seeking interpretation of and counsel about the results. However, most primary care physicians (PCPs) are not trained to interpret and communicate about DTC-PGT results. New guidelines must be developed to help PCPs maximize the potential of emerging DTC-PGT technologies.

The early 2000s saw unprecedented improvements in genotyping technology and analysis: the human genome sequence (Human Genome Project) and the cataloguing of human genetic variation (International HapMap Project) were completed. Altogether, these discoveries led to large-scale, genome-wide association studies and the subsequent identification of genetic variants associated with the risk of common complex diseases [1-3]. These advances enabled the introduction of direct-to-consumer personal genomic testing (DTC-PGT), which refers to a type of genetic test a consumer can purchase and complete without a referral from a health care professional. Interest in these tests skyrocketed through 2016, 2017, and 2018. In fact, figures reported in 2017 and 2018 showed that the number of people opting for a DTC-PGT in each of those years was higher than that in all of the previous years combined [4]. By mid-2019, it was estimated that over 26 million people had purchased a test from the leading DTC-PGT companies. Currently, a health and ancestry test by the company 23andMe costs US $199; the company claims over 10 million customers, most of whom are in the United States [5]. Although purchases leveled off in 2019, these figures from recent years are impressively high and suggest that, at present, roughly 1 in 13 Americans may have access to their personal genetic data via DTC-PGT.

Primary consumer motivations for seeking DTC-PGT are ancestry, health information, and curiosity [6]. Following the regulatory approvals issued by the Food and Drug Administration in 2017 and 2018, it has become more common for DTC-PGT to bundle ancestry information with health information [7]. Interpretating health data can be a convoluted process, especially when undertaken by the consumer without the participation or input of a health care professional. For example, since the majority of common diseases are polygenic, the presence of gene variants known to be associated with a disease does not necessarily manifest as clinical symptoms; heritable diseases have variable penetrance (eg, where patients may only have minor signs and symptoms). Moreover, there is the issue of questionable accuracy of these tests. One study found that 40% of genetic variants reported in the DTC-PGT raw data were false positives—a high rate that would be unacceptable in clinical laboratories [8]. Finally, tests might be performed incorrectly or in an unaccredited, uncertified lab; the case of Theranos [9] is a recent reminder that even clinical laboratories are not immune to compromised quality of science.

Consumers often turn to their primary care physicians (PCPs) for help in interpreting their DTC-PGT health data and finding meaning in the test results [6,10-12]. The PCP is put in a challenging position because they did not order the test, the test may have limited clinical validity, and they may not have enough knowledge to interpret it or provide advice. The result is a potential quagmire of a clinic visit where the PCP needs to navigate these unsolicited test results while still providing compassionate care for the patient. The number of tests purchased from 23andMe alone, together with the existing data on rates of sharing DTC-PGT results with physicians, indicates that a large number of PCPs are likely to encounter patients with their DTC-PGT results. The mean rate of people sharing DTC-PGT results with their PCPs is 27% as per the existing research [6,11,12]. At this rate, if 80% of 23andMe’s 10-million customer base are from the United States, it follows that about 2,080,000 people have already shared DTC-PGT results with their PCP. As of March 2019, the number of practicing PCPs in the United States was reported to be 479,856 [13]. On average, approximately 4 (precisely, 4.3) DTC-PGT test results reports are shared with each PCP, and this is likely an underestimate since it does not account for results from other companies (eg, Ancestry.com) or interpretation services.

Practicing PCPs are underprepared for this situation, and at present, there is no educational infrastructure in place to equip the next generation of PCPs. A study of 130 PCPs found that, although 88% had heard of 23andMe, less than a quarter of those PCPs (23%) felt comfortable discussing genetic risk factors for common diseases [14]. Although tech start-ups enthusiastically embraced genetics, medical education institutions were—and continue to be—lagging participants. There is still no widely accepted approach to genomics education. Some medical schools are starting to incorporate more genetic content into the curriculum [15], but the recipients of these lessons are vastly outnumbered by physicians educated before the era of genomics. In 2017, the majority of PCPs were between the ages of 45-49 years, and over one-quarter of PCPs were older than 60 years [16]. Thus, most practicing PCPs were trained prior to the completion of the Human Genome Project. Moreover, studies suggest that, among PCPs, there is a knowledge deficit as well as a paucity of confidence [11,14,17]. Therefore, teaching genomics content in medical schools does not necessarily translate into the ability of a PCP to interpret or communicate about genetic data presented to them by a patient. Medical students require opportunities to put the content of their genetics education into practice as they transition from the classroom to the clinic.

In the era of genomic medicine, the volume and complexity of medical knowledge exceed the capabilities of individual physicians. Moreover, scenarios involving consumer genomics often present complex problems interlaced with questions of ethics and beneficence. The ethical aspects associated with DTC-PGT are extensive. They include issues such as persuasive advertising, which is normal in the world of marketing but not in medical communication; unintended psychological impacts such as anxiety and distress related to testing and results; and the potential for genetic discrimination. Additionally, there are concerns about ambiguous practices related to informed consent as well as the storage, use, and third-party sale of genomic data. It remains largely unknown if PCPs are aware of these ethical challenges and, if they are, how best to address them in the increasingly shortened duration of clinical visits. Referring patients with DTC-PGT to geneticists and genetic counselors appears to be an obvious solution, but this is impractical and undermined by patients’ preferences to first consult their PCPs. The impracticality lies in the sheer lack of geneticists in the United States. Although patient caseloads have increased (in one study, geneticists reported an average of 10.2 new patients per week), the number of geneticists has not increased in kind [18]. Altogether, these factors contribute to the added responsibility upon PCPs to interpret the DTC-PGT results and engage in meaningful communication about those results with patients.

To reduce the extent to which DTC-PGT encumbers PCPs, the development and implementation of best practice guidelines should be seriously considered. These guidelines would help orient PCPs toward an appropriate standard of providing compassionate counsel to patients who seek to understand and interpret their DTC-PGT results. Guidelines should emphasize both education and clinical practices. Genomics education should be further integrated into the programs of undergraduate medical education and continuing medical education, and should extend beyond the role of genetics in human pathologies. There should also be a focus on providing an understanding of differential test efficacy (eg, Sanger sequencing vs single nucleotide polymorphism genotyping); potential clinical utility; and the ethical, legal, and social issues surrounding DTC-PGT. In addition, the scenarios of a patient sharing their DTC-PGT results should be a part of the practice-based learning sessions, which would present an opportunity to help students learn how to communicate with patients who bring complex personal data to the clinical encounter. The incorporation of active learning elements across the 4 years of medical school is critical in ensuring that students can carry their understanding of genomics and precision medicine education from didactic to clinical environments.

Guidelines on clinical practice should be consistent with the larger archetypal shift from paternalistic medicine to patient-centered care, patient autonomy, and shared decision making. The crux of guidelines should be how to effectively communicate and engage in dialogue surrounding DTC-PGT. A patient ordering their own test and bringing the results to their PCP disrupt the system in which most PCPs were trained [19]. Thus, the guidance on helping PCPs navigate their patients’ self-ordered, complex genetic information is of paramount importance. For the patient, the test may be less about genetic nuance and more about understanding their own story [20]. Finally, recommendations regarding DTC-PGT need to be mindful of the fact that physicians are currently in the midst of a burnout epidemic [21]. Burnout not only hinders the quality of patient care but also relinquishes the luxury of time and stymies enthusiasm to keep up with medical advances.

In 2019, Ancestry, a DTC-PGT provider company, awarded US $1 million to UpToDate—an organization that produces evidence-based information for clinical decision support systems [22]. This money was intended to aid the creation of content that assists physicians. Although well-intentioned, this initiative is not likely to be sufficient. Physicians need additional guidance regarding how to address their patients’ DTC-PGT data as well as other consumer testing devices and health information. The trends of consumer interest and adoption of such genetic health tests and devices are on an upward trajectory, and such guidance is the key to maximizing the benefit of and minimizing the burden from these emerging consumer-focused health technologies.

## Abbreviations

DTC-PGT: direct-to-consumer personal genomic testing
PCP: primary care physician
